# When knowledge is not enough: infection prevention and control practices and occupational biological exposures among dental professionals in Zimbabwe

**DOI:** 10.3389/fpubh.2026.1933281

**Published:** 2026-08-14

**Authors:** Tanusha Singh, Kundai Mavis Makozho

**Affiliations:** Department of Environmental Health, Faculty of Health Sciences, University of Johannesburg, Johannesburg, South Africa

**Keywords:** IPC, IPC—infection prevention and control, occupational exposure, occupational infection, risk control, sharps injury

## Abstract

**Background:**

Dental professionals are routinely exposed to occupational biological hazards, yet evidence from low-resource settings remains limited. This study assessed infection prevention and control (IPC) knowledge and practices, quantified occupational biological exposures, and identified factors associated with sharps injuries and occupational infections.

**Methods:**

A cross-sectional analytical study was conducted among 220 dental professionals in Bulawayo, Zimbabwe. Data were collected using a structured self-administered questionnaire and analysed using descriptive statistics and bivariate and multivariable logistic regression. Adjusted odds ratios (aORs) with 95% confidence intervals (CIs) were reported.

**Results:**

Lifetime sharps injury was reported by 61.2% of participants, 14.7% reported a sharps injury during the preceding 12 months, and 18.3% reported an occupational infection. Despite high awareness of core IPC principles, occupational exposure remained common. Recent sharps injury was independently associated with early-career role (aOR = 4.01; 95% CI: 1.26–12.76), eye protection use (aOR = 2.88; 95% CI: 1.21–6.84), poor hand hygiene adherence (aOR for often/always = 0.34; 95% CI: 0.12–0.97), and lower perception scores (aOR = 0.49 per unit increase; 95% CI: 0.32–0.75). Public-sector employment was independently associated with self-reported occupational infection (aOR = 2.66; 95% CI: 1.25–5.64).

**Conclusion:**

Dental professionals in Bulawayo experienced substantial occupational biological hazards despite generally high awareness of IPC principles. These findings demonstrate that IPC knowledge alone is insufficient to prevent occupational exposure. Strengthening competency-based IPC training, post-exposure management, institutional IPC systems, and targeted support for early-career dental professionals can reduce occupational injuries and occupational infections.

## 1 Introduction

Dental professionals are routinely exposed to biological hazards, through contact with blood, saliva, bioaerosols, contaminated surfaces, sharp instruments, and instrument reprocessing systems (1, 2). These exposures increase the risk of percutaneous injuries, mucocutaneous contamination, and transmission of pathogens including hepatitis B virus, hepatitis C virus, human immunodeficiency virus (HIV), tuberculosis, influenza and other respiratory infections. IPC is the primary risk-control strategy for preventing occupational biological exposures in dental practice (3–6).

Implementation of IPC measures, however, is often constrained in low- and middle-income countries (LMICs) by shortages of personal protective equipment (PPE), inadequate sterilisation resources, high patient workloads, outdated protocols, and limited continuing professional education (7, 8). Previous studies have identified deficiencies in IPC knowledge and practices, including inconsistent use of eye protection, inadequate sterilisation procedures, unsafe post-exposure management, and weak institutional IPC systems (9–13). Most previous studies have described IPC knowledge, attitudes, and practices in isolation. Far fewer have examined whether IPC knowledge translates into reduced occupational biological exposures or identified the workplace factors associated with occupational injuries and infections. This implementation gap limits the ability of healthcare organisations to develop evidence-informed IPC programmes (14). Moreover, health behavior theories, including the Health Belief Model (HBM), suggest that IPC compliance is influenced by perceived susceptibility, perceived severity, perceived benefits, and perceived barriers to preventive practices (15–17). This study assessed IPC knowledge and practices, quantified occupational biological exposures, and identified behavioral and workplace factors associated with recent sharps injuries and occupational infections among dental professionals in Zimbabwe. The analysis was framed from an occupational safety and health (OSH) perspective, with IPC treated as a risk-control system that operates through individual behavior, training, facility resources, and institutional safety culture.

## 2 Materials and methods

### 2.1 Study design and setting

A cross-sectional analytical study was conducted among dental professionals practicing in Bulawayo, Zimbabwe. Bulawayo includes public, private, parastatal, and church-owned (mission) dental facilities serving urban and peri-urban populations.

### 2.2 Study population and eligibility criteria

The population comprised dental professionals aged ≥18 years engaged in clinical or dental laboratory work in Bulawayo during the study period. Eligible participants included trainee dental assistants, intern dentists, dental therapists, hygienists, dental technicians, general dentists, orthodontists, and other personnel involved in clinical dental service delivery. Administrative and other non-clinical staff, and dental professionals practicing outside Bulawayo, were excluded.

### 2.3 Sample size and sampling

The minimum sample size was calculated using Cochran’s formula for cross-sectional studies, assuming a 95% confidence level, 5% margin of error, and a conservative 50% prevalence estimate for adequate IPC knowledge in the absence of local data. Following finite population correction, the minimum required sample was 110 participants. A total of 220 participants were recruited from public, private, parastatal, and mission facilities using availability-based convenience sampling. Although facilities were selected to maximise representation across institutional types, convenience sampling is acknowledged as a limitation because of its potential impact on representativeness.

### 2.4 Data collection

Data were collected from August to October 2022 using a structured, self-administered questionnaire adapted from IPC guidelines and validated KAP instruments used in dental settings (18–20). The questionnaire collected information on socio-demographic and occupational characteristics, IPC knowledge, perceptions of barriers and risks, IPC practices, history of sharps injury, and self-reported occupational infection. It was administered in English, took approximately 20–30 min to complete, and collected no personal identifying information.

### 2.5 Validity and reliability

Content and face validity were established through expert review by two senior dental professionals and a biostatistician to ensure alignment with the study objectives, current IPC guidelines, and relevant literature. The questionnaire was piloted among ten dental professionals in Gweru to assess clarity, comprehensibility, and reliability, with feedback informing minor wording revisions. Reliability was assessed using Cronbach’s alpha. During pilot testing, the perception and practice scales demonstrated acceptable internal consistency (*α* = 0.74 and *α* = 0.80, respectively), whereas the knowledge scale showed lower internal consistency (*α* = 0.45). Item analysis informed refinement of the knowledge items before administration of the final questionnaire, which demonstrated good overall internal consistency (Cronbach’s *α* = 0.83) (11).

### 2.6 Variables

The primary occupational exposure outcomes were (1) sharps injury during the preceding 12 months, coded as none versus one or more injuries, and (2) self-reported occupational infection, coded as yes/no. Lifetime sharps injury was analysed descriptively to distinguish cumulative career exposure from recent occupational injury. Professional category was grouped into six categories, with the single orthodontist classified as ‘Other’. Early-career role comprised intern dentists and trainee dental assistants because both represent supervised practitioners with limited clinical experience. Public facility status was coded as government/public versus all other facility types. IPC knowledge, perception, and practice composite scores were analysed as continuous variables. Higher perception scores indicated stronger perceptions of IPC-related barriers and the importance of risk control, whereas higher practice scores reflected better self-reported IPC practices. For selected item-level analyses, correct knowledge responses were defined according to IPC guidance; for negatively worded items, correct responses were disagreement or strong disagreement with the false statement.

### 2.7 Data management and statistical analysis

Data were cleaned and coded using Microsoft Excel (Microsoft Corporation, Redmond, WA, USA) and analysed using IBM SPSS Statistics for Windows, Version 27.0 (IBM Corp., Armonk, NY, USA). Categorical variables were summarised using frequencies and percentages, while continuous variables were summarised using means and standard deviations. Bivariate logistic regression was used to estimate crude odds ratios (ORs) for recent sharps injury and occupational infection. Multivariable logistic regression analyses were performed to identify factors independently associated with recent sharps injury and self-reported occupational infection. Variables with *p* < 0.20 in bivariate analyses, together with epidemiologically relevant covariates identified *a priori*, were entered into multivariable logistic regression models. Adjusted odds ratios (aORs) and 95% confidence intervals (CIs) were estimated. Statistical significance was set at *p* < 0.05.

### 2.8 Ethical considerations

Ethical approval was obtained from the University of Johannesburg Faculty of Health Sciences Research Ethics Committee (REC-3356-2025) and the Medical and Dental Practitioners Council of Zimbabwe (REF: CB/fc/c02/06/24). Written informed consent was obtained from all participants. Data were stored securely with access restricted to the research team.

## 3 Results

A total of 220 dental professionals participated with a mean age of 35.21 years (SD = 8.61), and the mean duration of practice was 7.66 years (SD = 6.17) (Table 1). Participants were almost evenly distributed by sex, with 112 females (50.9%) and 103 males (46.8%). General dentists comprised the largest professional group (29.1%), and half worked in private facilities (50.0%).

**Table 1:** Socio-demographic and occupational characteristics of participants (*N* = 220).

Variable	Frequency (*n*)	Percent (%)
Age (years) (mean, SD)	35.21	8.61
Sex		
Male	103	46.8
Female	112	50.9
Not reported	5	2.3
Highest level of education		
O Level	46	20.9
A Level	13	5.9
Diploma	58	26.4
Bachelor’s Degree	63	28.6
Master’s Degree	15	6.8
PhD	1	0.5
Other	24	10.9
Professional category		
Trainee dental assistant	15	6.8
Intern dentist	11	5.0
Dental therapist/hygienist	30	13.6
Dental technician	24	10.9
General dentist	64	29.1
Orthodontist	1	0.5
Other	75	34.1
Facility type		
Government	79	35.9
Private	110	50.0
Parastatal	12	5.5
Church-owned	17	7.7
Other	2	0.9
Years in practice (mean, SD)	7.66	6.17

SD, standard deviation.

### 3.1 Occupational exposure outcomes

Lifetime sharps injury prevalence was 61.2% (134/219), while 14.7% (32/217) reported at least one sharps injury during the preceding 12 months (Table 2). Self-reported occupational infection was reported by 18.3% (40/219). Recent sharps injuries were most common among intern dentists and trainee dental assistants, whereas occupational infections were highest among intern dentists.

**Table 2:** Occupational exposure outcomes by professional category.

Professional category	Sharps injury, past 12 months		Self-reported occupational infection	
	*n*/*N*	Percent (%)	*n*/*N*	Percent (%)
Intern dentist	3/11	27.3	3/11	27.3
Trainee dental assistant	3/14	21.4	3/15	20.0
General dentist	11/64	17.2	12/64	18.8
Dental therapist/hygienist	4/29	13.8	4/30	13.3
Dental technician	1/24	4.2	3/24	12.5
Other	10/75	13.3	15/75	20.0
Overall	32/217	14.7	40/219	18.3

*n*, number of participants reporting the outcome; *N*, total number of participants with available data for that professional category or outcome.

### 3.2 Infection prevention and control knowledge

Participants demonstrated high awareness of core IPC principles, although important implementation-related gaps in knowledge remained (Table 3). More than 90% recognised that IPC protects both patients and dental professionals, while 75.5% correctly rejected the false statement that a needle-stick injury should be washed with bleach. Nevertheless, one-quarter of participants either believed this incorrect practice or were uncertain. Only 55.0% correctly recognised that two doses of hepatitis B vaccine are insufficient for immunity.

**Table 3:** Key IPC knowledge indicators among dental professionals.

Knowledge indicator	Correct response, *n* (%)	Item
		Mean (SD)
IPC prevents infection transmission	205 (93.2)	4.43 (0.97)
Infections can be transmitted through splatter/splash	200 (91.3)	1.62 (0.98)
Infections can be transmitted through inhalation	194 (88.2)	1.69 (1.02)
Infections can be transmitted through skin contact	166 (75.5)	3.89 (1.00)
Needle-stick injury site should not be washed with bleach	166 (75.5)	2.10 (1.11)
Dental handpieces/scalers should not be sterilised only once daily	136 (61.8)	2.48 (1.42)
Good IPC protects patients and dental professionals	213 (96.8)	4.68 (0.68)
Two hepatitis B vaccine doses are not sufficient for immunity	121 (55.0)	2.75 (1.33)

*n*, number of participants; %, percentage; SD, standard deviation.

### 3.3 Infection prevention and control practices and composite scores

Self-reported adherence was high for masks, gloves and hand hygiene, but lower for written IPC guideline use, written sharps-injury protocol use, and sterilisation wrapping bags and eye protection (Table 4). Composite IPC knowledge, perception and practice scores are presented in Table 5. Mean perception scores were highest (4.46 ± 0.86), followed by practice (3.88 ± 0.79), whereas knowledge scores were lower (2.44 ± 1.07).

**Table 4:** Key IPC practice indicators among participants.

Practice indicator	Always/often	Item
	*n* (%)	Mean (SD)
Written IPC guideline/manual always used	61 (27.7)	3.62 (1.17)
Sterilisation wrapping bags always used	66 (30.3)	3.67 (1.18)
Written sharps-injury protocol always used	77 (35.2)	3.65 (1.28)
Hand hygiene often/always before and after procedures	195 (89.0)	4.42 (0.69)
Gloves are always worn during procedures	184 (83.6)	4.73 (0.67)
Masks always worn during procedures	203 (92.3)	4.88 (0.46)
Eye protection always worn during procedures	84 (38.2)	4.02 (0.93)
Sharps disposed in designated container	128 (58.4)	4.36 (0.88)

*n*, number of participants; %, percentage; SD, standard deviation.

**Table 5:** Composite IPC knowledge, perception and practice scores.

Composite score	*n*	Mean (SD)	Median	Range
Knowledge	220	2.44 (1.07)	2.33	1.00–5.00
Perceptions	220	4.46 (0.86)	4.83	1.17–5.00
Practices	220	3.88 (0.79)	3.90	1.20–5.00

### 3.4 Factors associated with occupational exposure outcomes

Table 6 presents the bivariate analyses. Higher IPC perception scores were associated with lower odds of recent sharps injury (OR = 0.56, 95% CI: 0.40–0.79; *p* = 0.001). Eye protection use, hand hygiene, and written IPC guideline use showed borderline associations (*p* = 0.052–0.056). No significant associations were observed for early-career role, public facility employment, knowledge score, or practice score. Public facility employment was the only factor associated with occupational infection (OR = 2.97, 95% CI: 1.47–6.00; *p* = 0.002).

**Table 6:** Bivariate logistic regression for occupational exposure outcomes.

Variable	Sharps injury OR (95% CI)	*p*-value	Self-reported occupational infection OR (95% CI)	*p*-value
Early-career role (intern dentist/trainee dental assistant)	2.02 (0.74–5.52)	0.172	1.40 (0.52–3.76)	0.500
Public facility	1.08 (0.50–2.35)	0.843	2.97 (1.47–6.00)	0.002
Eye protection always	2.09 (0.98–4.46)	0.056	1.40 (0.70–2.80)	0.340
Hand hygiene often/always	0.38 (0.15–1.01)	0.053	0.88 (0.31–2.51)	0.812
Knowledge score, per one-unit increase	1.21 (0.86–1.71)	0.273	1.09 (0.79–1.50)	0.591
Perception score, per one-unit increase	0.56 (0.40–0.79)	0.001	1.22 (0.77–1.93)	0.392
Practice score, per one-unit increase	0.82 (0.52–1.31)	0.409	0.68 (0.45–1.05)	0.079
Written IPC guideline always used	0.34 (0.11–1.01)	0.052	0.73 (0.32–1.64)	0.444

CI, confidence interval; IPC, infection prevention and control; OR, odds ratio. Per one-unit increase means that the odds ratio represents the change in the odds of the outcome associated with a 1-point increase in the composite score.

**Figure 1:**
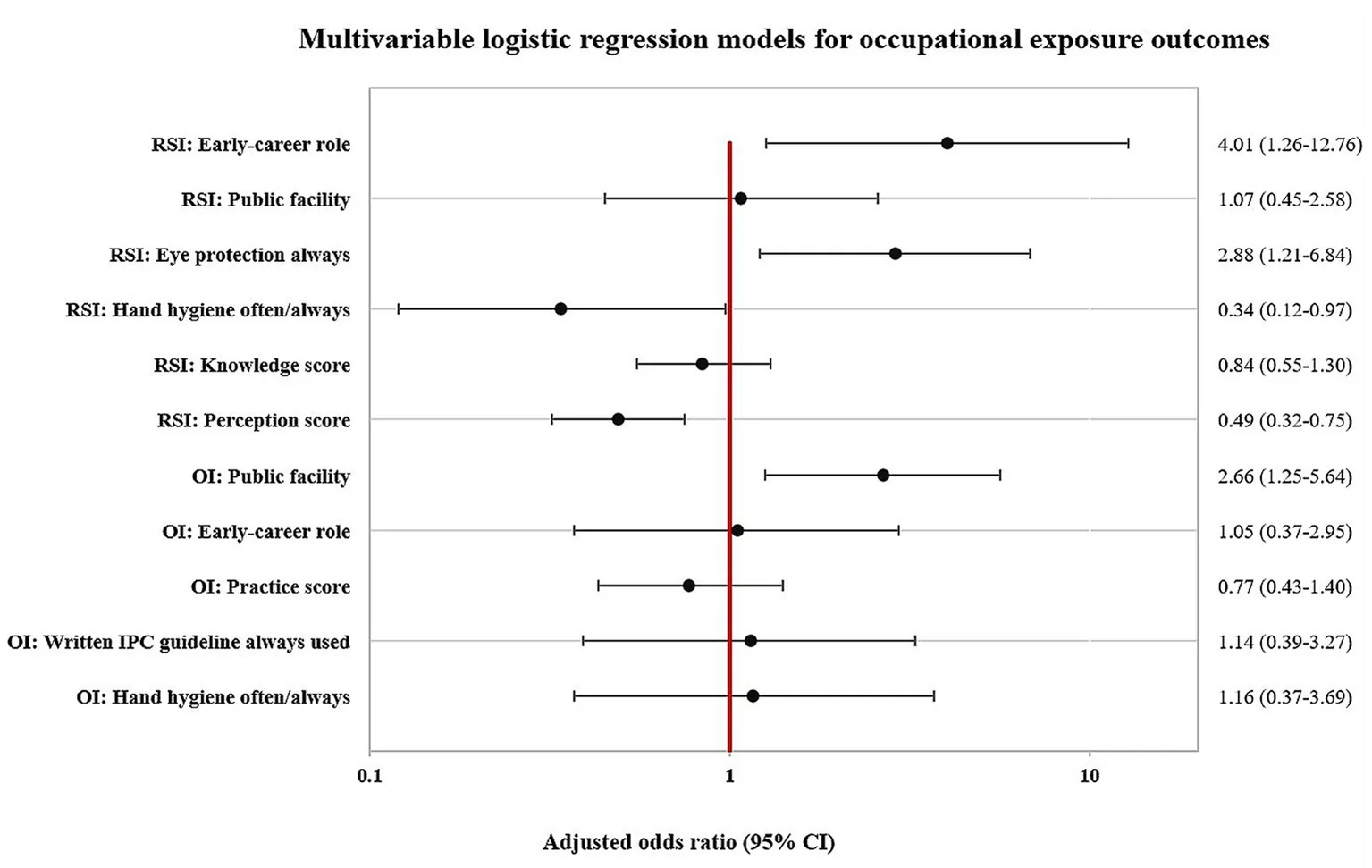
Adjusted odds ratios (95% CIs) from multivariable logistic regression models of recent sharps injury and self-reported occupational infection. Models included variables with *p* < 0.20 in bivariate analyses and epidemiologically relevant covariates. Both models demonstrated good fit (Hosmer–Lemeshow *p* > 0.05) with no evidence of multicollinearity (all VIFs < 2).

Figure 1 presents the adjusted logistic regression models. Early-career role was independently associated with higher odds of recent sharps injury, whereas higher IPC perception scores and frequent hand hygiene were protective. Eye protection use remained positively associated with recent sharps injury. Public facility employment was the only independent predictor of occupational infection. Both models demonstrated good fit (Hosmer–Lemeshow *p* > 0.05), with no evidence of problematic multicollinearity (all variance inflation factors (VIFs) < 2).

## 4 Discussion

This study found that dental professionals in Bulawayo experienced substantial occupational biological hazards. Approximately six in ten participants reported a lifetime sharps injury, and approximately one in seven reported at least one sharps injury in the preceding 12 months. Nearly one in five reported self-reported occupational infection. These findings indicate that occupational exposures remain a substantial workplace health challenge despite generally high awareness of IPC principles. Unlike many previous studies that have focused primarily on describing IPC knowledge or compliance, this study linked IPC indicators with measurable occupational outcomes, including recent sharps injuries and self-reported occupational infections. This provides insight into the IPC-related factors most strongly associated with occupational risk and highlights that high awareness alone does not necessarily translate into safer clinical practice.

The distinction between lifetime and recent sharps injury is important. Lifetime injury reflects cumulative occupational exposure across the career course, whereas past-12-month injury is a more direct indicator of current workplace risk. The high lifetime prevalence suggests that sharps injury is embedded in routine dental work, while the recent injury prevalence shows that active prevention gaps remain. For occupational safety and health systems, this means that interventions should not rely solely on initial professional training; they require sustained supervision, reporting systems, post-exposure protocols and periodic competency-based refresher training. Beyond documenting the burden of occupational exposure, this study identified several behavioral and workplace determinants that may inform prevention strategies.

Early-career professional status was independently associated with recent sharps injury. Intern dentists and trainee dental assistants may have higher exposure because they are still developing procedural fluency, situational awareness and confidence in managing sharp instruments under time pressure. This finding supports targeted supervision and simulation-based training for early-career personnel, especially during procedures involving needles, scalers, burs and contaminated instruments. Similar findings have been reported internationally, where occupational injury rates decline with increasing clinical experience and procedural competence (14, 18, 21).

The high self-reported compliance with gloves and masks aligns with standard precautions (22, 23), suggesting that basic IPC principles are well recognised. However, substantially lower eye protection use highlights an important gap in adherence to recommended IPC practices, leaving practitioners vulnerable to splashes and aerosols (24–26). Although this study did not specifically assess pandemic-related changes, the widespread use of respiratory protection during the COVID-19 pandemic may have also contributed to sustained mask use (27). However, persistent deficiencies in eye protection, instrument reprocessing knowledge, and post-exposure management indicate that comprehensive occupational IPC systems remain incompletely institutionalised.

Higher perception scores were protective against recent sharps injury. This suggests that greater awareness of occupational hazards and perceived susceptibility may influence safer behavior (28). However, knowledge scores were not independently associated with sharps injury, indicating that knowledge alone is insufficient. Occupational health interventions should therefore go beyond information transfer towards competency-based training, supportive supervision, workflow redesign, systems strengthening, and reinforcement of safety culture (29).

Unexpectedly, consistent eye protection use was independently associated with higher odds of recent sharps injury. This finding is unlikely to reflect a harmful effect of eye protection itself but more plausibly greater exposure to invasive or high-risk clinical procedures among practitioners who routinely use eye protection. Higher procedure intensity and patient contact may therefore increase both the likelihood of eye protection use and the risk of sharps injury. These findings highlight the limitations of cross-sectional analyses and the need for future studies to account for procedure mix, patient volume, and task-specific exposure intensity.

Higher IPC perception scores were independently associated with lower odds of recent sharps injury, suggesting that greater awareness of occupational risk may promote safer workplace behaviors, consistent with behavioral theories of risk perception (28). However, positive perceptions alone are unlikely to ensure safe practice where structural barriers, including limited PPE, sterilisation resources, and institutional support, persist. Knowledge gaps in instrument reprocessing were evident, particularly regarding sterilisation of dental handpieces and ultrasonic scalers (30). These instruments, classified as critical items, require sterilisation after each use, and failure to do so poses a significant risk of cross-contamination (15, 16). Although most participants correctly rejected washing a needle-stick injury with bleach, approximately one-quarter either believed this incorrect practice or were uncertain, indicating persistent gaps in post-exposure management knowledge (16). Knowledge of hepatitis B vaccination requirements was also suboptimal, with only just over half of participants correctly identifying the recommended vaccination schedule. These findings suggest deficiencies in standardised training on occupational exposure management, consistent with previous African and international studies of dental IPC practices (31, 32).

The broader context of Zimbabwe’s resource-limited healthcare system must also be considered in interpreting these findings. Constraints in PPE and sterilisation supply chains, heavy patient loads in public facilities, and outdated national IPC guidelines create a challenging environment for maintaining high-level infection control (18). The identified deficiencies likely reflect systemic rather than individual factors, including gaps in pre-service and in-service training on hepatitis B vaccination and instrument reprocessing (19).

Public-sector employment was independently associated with self-reported occupational infection. This may reflect higher patient loads, constrained resources, shortages of PPE and sterilisation supplies, and weaker institutional IPC systems in public facilities. The findings should be interpreted cautiously because occupational infection was self-reported and not laboratory confirmed. Nevertheless, they highlight the need to strengthen facility-level IPC and occupational health systems in resource-constrained dental services. Differences in patient volume and case-mix between public and private facilities may also have contributed to this association.

The item-level findings identify practical priorities for intervention. Misconceptions regarding bleach use following needle-stick injury and limited understanding of hepatitis B vaccination requirements are particularly concerning because they directly affect post-exposure management and prevention of blood–borne infections. Limited use of written IPC guidelines and sharps injury protocols further suggests reliance on individual practice rather than institutional IPC systems. From an OSH perspective, these protocols should be visible, accessible, regularly rehearsed, and integrated into reporting and post-exposure care pathways. These findings reinforce that IPC in dentistry should be viewed as a core occupational health intervention rather than solely a patient-safety requirement. Reducing occupational biological hazards requires coordinated action at individual, organisational, and health-system levels through competency-based practice, reliable PPE and sterilisation resources, safe sharps management, effective post-exposure protocols, and strong institutional accountability. Strengthening these systems will improve both healthcare worker safety and the quality of patient care.

## 5 Strengths and limitations

The study’s strengths include the integration of IPC knowledge and practices with measurable occupational biological exposure outcomes, inclusion of multiple dental professional categories and facility types, and analysis of both behavioral and workplace determinants. Rather than describing IPC knowledge alone, the study evaluated its relationship with occupational risk by identifying factors associated with recent sharps injuries and occupational infections. To our knowledge, this is among the first studies from Zimbabwe to integrate IPC knowledge and practice with occupational biological exposure outcomes among dental professionals. The independent re-analysis of the dataset further strengthened the consistency of the statistical analyses, tables, and interpretation.

Several limitations should be considered. The cross-sectional design precludes causal inference. Outcomes and practices were self-reported and may be subject to recall and social desirability bias. Self-reported occupational infection was not clinically verified and may have included infections acquired outside the dental setting. Convenience sampling may limit generalisability beyond participating facilities in Bulawayo, and small professional subgroups reduced the precision of some estimates. The multivariable model for recent sharps injury included only 32 events; therefore, the findings should be interpreted cautiously, although they remain policy-relevant. Future studies should incorporate incident reporting data, laboratory confirmation of occupational infections, procedure-level exposure measures, and prospective longitudinal designs.

## 6 Conclusion

Dental professionals in Bulawayo face substantial occupational biological hazards, including high lifetime and recent sharps injury rates and self-reported occupational infections. Early-career dental workers were at increased risk of recent sharps injury, while public-sector employment was associated with occupational infection. Despite high IPC awareness, occupational exposures remain common, demonstrating that knowledge alone is insufficient to prevent workplace biological hazards. Reducing these risks requires competency-based training, robust institutional IPC systems, reliable PPE and sterilisation resources, safe sharps management, occupational surveillance, and accessible post-exposure management. Embedding these measures within routine occupational health programmes will strengthen healthcare worker safety and improve the quality of patient care.

## Data Availability

The raw data supporting the conclusions of this article will be made available by the authors, without undue reservation.
