# Supplementary material for: When knowledge is not enough: infection prevention and control practices and occupational biological exposures among dental professionals in Zimbabwe
Source: Front Public Health. 2026 Aug 14;14:1933281. doi: 10.3389/fpubh.2026.1933281 (PMC13522109; doi:10.3389/fpubh.2026.1933281)
Supplement: Supplementary file 1 [file Table_1.DOCX]

Supplementary Material

# 1 Supplementary Data

Personal and workforce pressures were also widely reported: 86.3% of participants agreed or strongly agreed that stress, anxiety, fatigue or burnout affected their response, and 80.3% reported challenges associated with prolonged face-mask use. In contrast, only 19.6% considered mental health support adequate and 7.6% considered financial compensation adequate (Table S1).

**Supplementary Table S1. Perceived adequacy and personal/workplace factors.**

| **Perception item** | **Strongly disagree n (%)** | **Disagree n (%)** | **Neutral n (%)** | **Agree n (%)** | **Strongly agree n (%)** | **Mean (SD)** |
| --- | --- | --- | --- | --- | --- | --- |
| Adequate vaccination | 0  (0%) | 51 (11.1%) | 78 (17.0%) | 204 (44.3%) | 127 (27.6%) | 2.88 (0.94) |
| Adequate training | 40  (8.7%) | 92 (20.0%) | 96 (20.9%) | 232 (50.4%) | 0  (0.0%) | 2.13 (1.02) |
| Adequate PPE | 24  (5.2%) | 132 (28.7%) | 127 (27.6%) | 177 (38.5%) | 0  (0.0%) | 1.99 (0.94) |
| Adequate clinical resources | 0  (0%) | 76 (16.5%) | 115 (25.0%) | 205 (44.6%) | 64  (13.9%) | 2.56 (0.93) |
| Personal factors | 28  (6.1%) | 35  (7.6%) | 0  (0.0%) | 160 (34.8%) | 237 (51.5%) | 3.18 (1.16) |
| Challenges with face mask use | 0 (0%) | 60 (13.0%) | 31  (6.7%) | 182 (39.6%) | 187 (40.7%) | 3.08 (1.00) |
| Adequate medication | 51 (11.1%) | 104 (22.6%) | 120 (26.1%) | 134 (29.1%) | 51 (11.1%) | 2.07 (1.18) |
| Adequate mental health counselling | 120 (26.1%) | 152 (33.0%) | 98 (21.3%) | 90 (19.6%) | 0  (0.0%) | 1.34 (1.07) |
| Adequate financial compensation | 178 (38.7%) | 170(37.0%) | 77 (16.7%) | 35  (7.6%) | 0  (0.0%) | 0.93 (0.93) |

**Abbreviations:** n, number of participants; PPE, personal protective equipment; SD, standard deviation. Percentages are based on N=460. Perception items were rated on a five-point Likert scale from strongly disagree to strongly agree. Personal factors included stress, anxiety, fatigue and burnout.
